# Supplementary material for: Active site specificity profiling datasets of matrix metalloproteinases (MMPs) 1, 2, 3, 7, 8, 9, 12, 13 and 14
Source: Data Brief. 2016 Feb 22;7:299–310. doi: 10.1016/j.dib.2016.02.036 (PMC4777984; doi:10.1016/j.dib.2016.02.036)
Supplement: Supplementary file 10 — Supplementary material [file mmc10.zip › WebPICS_hMMP8_G_1%/P2.html]

 

PICS results


|  |  |
| --- | --- |
| **P2\_A**  14 in 120 sites   11.7 %    effects > 10 perc. pnts.  (vice-versa in brackets)  P3\_V: 10.6 (11.4)   P1\_A: 10.6 (11.4)   P1\_Q: 12.2 (15.6)   P1prime\_Q: 18.6 (21.6)   P1prime\_Y: 11.0 (38.3)   P3prime\_S: 11.4 (13.3) |  |
| **P2\_F**  9 in 120 sites   7.5 %    effects > 10 perc. pnts.  (vice-versa in brackets)  P1\_Q: 35.2 (28.9)   P1prime\_F: 16.4 (21.1)   P1prime\_V: 23.3 (17.5)   P2prime\_I: 22.5 (15.6)   P2prime\_V: 20.0 (11.3)   P3prime\_N: 25.0 (22.5) |  |
| **P2\_H**  3 in 120 sites   2.5 %    effects > 10 perc. pnts.  (vice-versa in brackets)  P1prime\_Y: 30.0 (22.5)   P3prime\_Q: 27.5 (11.8) |  |
| **P2\_K**  11 in 120 sites   9.2 %    effects > 10 perc. pnts.  (vice-versa in brackets)  P3\_P: 31.2 (12.2)   P1\_A: 34.7 (29.3)   P2prime\_K: 33.7 (14.8) |  |
| **P2\_L**  16 in 120 sites   13.3 %    effects > 10 perc. pnts.  (vice-versa in brackets)  P3\_I: 17.5 (31.1)   P1\_N: 18.3 (12.8)   P1\_P: 11.3 (20.0)   P1prime\_C: 13.0 (29.6) |  |
| **P2\_M**  4 in 120 sites   3.3 %    effects > 10 perc. pnts.  (vice-versa in brackets)  P1prime\_V: 40.0 (13.4) |  |
| **P2\_N**  6 in 120 sites   5.0 %    effects > 10 perc. pnts.  (vice-versa in brackets)  P3\_V: 22.5 (10.4) |  |
| **P2\_Q**  12 in 120 sites   10.0 %    effects > 10 perc. pnts.  (vice-versa in brackets)  P3prime\_Q: 19.2 (32.9) |  |
| **P2\_W**  4 in 120 sites   3.3 %    effects > 10 perc. pnts.  (vice-versa in brackets)  P3\_H: 70.8 (56.7)   P1\_P: 67.5 (30.0)   P1prime\_F: 69.2 (39.6)   P2prime\_Q: 90.0 (30.0)   P3prime\_V: 66.7 (26.7) |  |
| **P2\_Y**  8 in 120 sites   6.7 %    effects > 10 perc. pnts.  (vice-versa in brackets)  P1prime\_C: 19.2 (21.9) |  |
